# Presenilin-dependent intramembrane cleavage of ephrin-B1

**DOI:** 10.1186/1750-1326-1-2

**Published:** 2006-06-12

**Authors:** Taisuke Tomita, Sayaka Tanaka, Yuichi Morohashi, Takeshi Iwatsubo

**Affiliations:** 1 Department of Neuropathology and Neuroscience, Graduate School of Pharmaceutical Sciences, The University of Tokyo, 7-3-1 Hongo, Bunkyo-ku, Tokyo, 113-0033, Japan

## Abstract

**Background:**

Presenilin-dependent γ-secretase cleavage of several transmembrane proteins, including amyloid-β precursor protein and Notch, mediates the intramembrane proteolysis to liberate their intracellular domains that are involved in cellular signaling. Considering γ-secretase inhibitors as therapeutics for Alzheimer's disease, understanding the physiologically and biologically important substrate for γ-secretase activity in brains is emerging issue. To elucidate the molecular mechanism and physiological role of γ-secretase, we screened candidate molecules for γ-secretase substrates.

**Results:**

We show that ephrin-B1, that participates in cell-cell repulsive and attractive signaling together with its Eph receptor, constitutively undergoes ectodomain shedding and that the residual membrane-tethered fragment is sequentially cleaved by γ-secretase to release the intracellular domain. Furthermore, overexpression of membrane-tethered ephrin-B1 caused protrusion of numerous cellular processes consisted of F-actin, that required the preservation of the most C-terminal region of ephrin-B1. In contrast, soluble intracellular domain translocated into the nucleus and had no effect on cell morphology.

**Conclusion:**

Our findings suggest that ephrin-B is a genuine substrate for γ-secretase and regulates the cytoskeletal dynamics through intramembrane proteolysis.

## Background

Alzheimer disease (AD) is a neurodegenerative disorder characterized pathologically by neuronal loss in the cerebral cortex accompanied by the deposition of amyloid β peptides (Aβ) as senile plaques. Aβ is produced by sequential proteolytic cleavages of the amyloid-β precursor protein (APP) by a set of membrane-bound proteases termed β- and γ-secretases. γ-Secretase is an unusual aspartic protease that cleaves APP within the transmembrane domain (TMD) [1]. Presenilins (PS) are highly conserved polytopic transmembrane proteins that are mutated in a majority of pedigrees of early-onset familial Alzheimer's disease. PS represent the active site component of γ-secretase, a multiprotein complex comprised of Nicastrin, APH-1 and PEN-2 [2]. FAD-linked mutations in PS genes cause an increase in the production of Aβ ending at position 42, that most readily form amyloid deposits in AD brains, implicating the seminal role of γ-secretase/PS complex in the pathogenesis of AD.

It has been shown that a number of type I single-span membrane proteins are cleaved by γ-secretase [3]. Although γ-secretase is unable to cleave the full-length (FL) form of these substrates, the membrane-tethered C-terminal fragments (CTF) generated by ectodomain shedding are processed by γ-secretase to liberate N-terminal small fragments and C-terminal intracellular domains (ICD) into luminal and cytoplasmic side, respectively. The liberated ICD translocates into the nucleus and participates in signal transduction (e.g., Notch [4]). Thus, the γ-secretase-mediated intramembrane proteolysis is highlighted as a novel mode of proteolysis-dependent signal transduction utilizing ICD [5]. Recently it was reported that the administration of functional γ-secretase inhibitors in rodents caused an alteration in lymphopoiesis and intestinal cell differentiation through inhibition of Notch signaling [1]. Thus, the understanding of the molecular mechanism of the unusual mode of intramembrane proteolysis is a critical problem for the development of APP-specific γ-secretase inhibitors for the treatment of AD.

Although the cleavage sites of some substrates have been identified, the amino acid sequences within the transmembrane domain that undergo γ-secretase cleavage exhibit a loose homology. To elucidate the molecular mechanism and physiological role of γ-secretase in brains, we screened candidate molecules for γ-secretase substrates using several criteria. Here we identified ephrin-B1 as a novel substrate for γ-secretase-mediated intramembrane proteolysis.

## Results

### Proteolytic processing of ephrin-B

**Figure 1:**
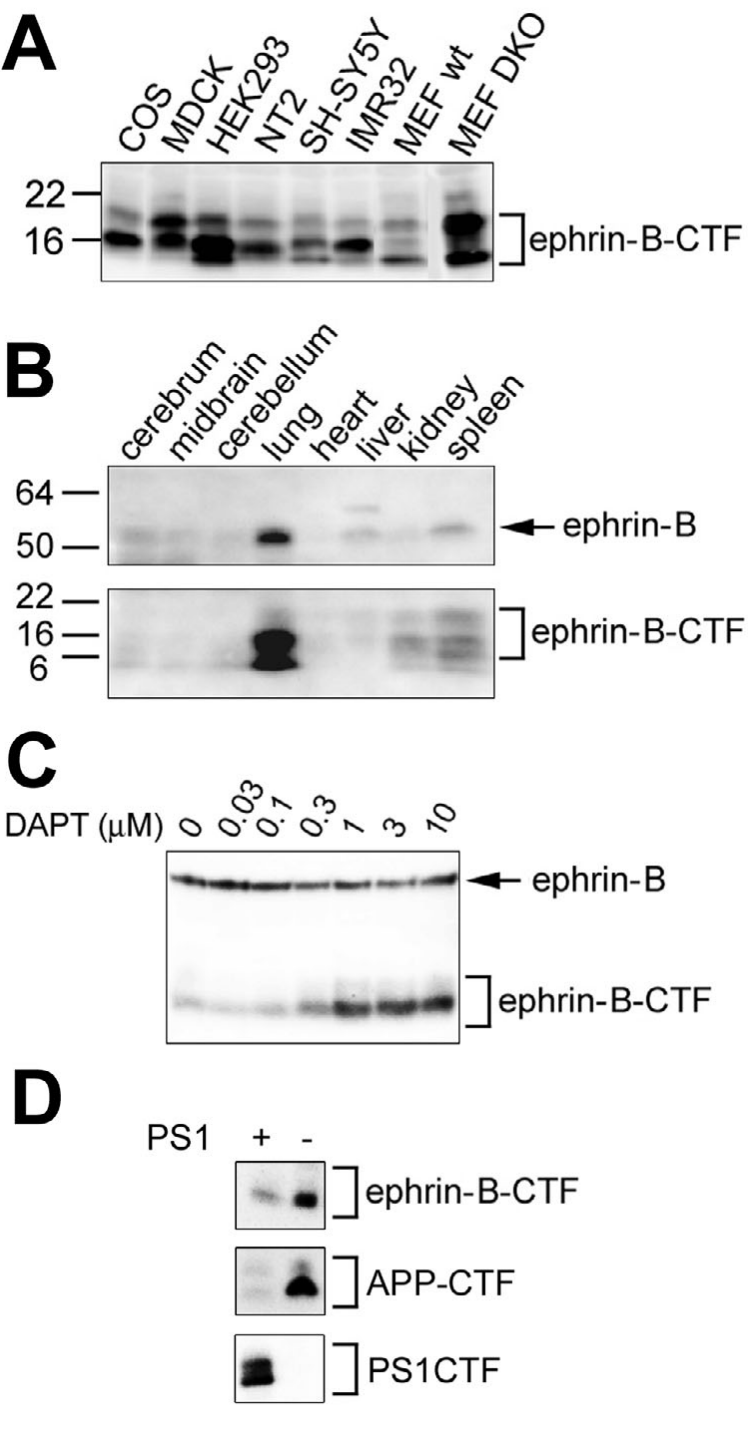
**Immunoblot analysis of endogenous ephrin-B CTFs**. Membrane fractions from cultured cells (A) and mouse organs (B) were subjected to immunoblot analyses using anti-ephrin-B antibody. C) Immunoblot analysis of lysates from COS cells treated with a γ-secretase inhibitor DAPT. D) Immunoblot analysis of lysates from DKO cells expressing PS1.

Although several transmembrane proteins are reported as a substrate for PS/γ-secretase-dependent intramembrane cleavage, a low homology of the amino acid sequences of transmembrane domain (TMD) has been found among these substrates [5]. We searched the database for novel γ-secretase substrates that suffice the characteristics of known substrates using following criteria: i) type I transmembrane protein, ii) carrying a receptor/ligand structure, iii) engaged in cell-cell interaction, iv) undergoes ectodomain shedding (or harboring a homologous sequence to other proteins undergoing shedding at juxtamembrane region) v) an accumulation of endogenous C-terminal fragment (CTF) in PS-depleted cells. We selected some candidate molecules and analyzed the membrane fractions from various cell lines including MEFs from *Psen1*^-/-^/*Psen2*^-/- ^(DKO) mice [6] by immunoblotting using commercially available antibodies against the C-terminal region. We found that an antibody against ephrin-B probed ~14–17 kDa bands corresponding to the membrane-tethered CTF in various cell membranes, in addition to ~40–50 kDa bands representing the endogenous full-length (FL) protein, and that these ~14–17 kDa bands were accumulated in membranes from PS DKO MEF (Fig. 1A). We also detected ~14–17 kDa bands that reacted with an anti-ephrin-B antibody in membrane fractions of adult mouse organs (Fig. 1B). Moreover, treatment by a γ-secretase inhibitor, DAPT, caused a concentration-dependent accumulation of endogenous ephrin-B-CTF in COS cells (Fig. 1C). Finally, the accumulation of ephrin-B CTF was abolished by the overexpression of PS1 in DKO cells (Fig. 1D). Taken together, these data raised the possibility that ephrin-B-CTF is processed by PS-dependent γ-secretase under a physiological condition, in a similar manner to known substrates for γ-secretase.

**Figure 2:**
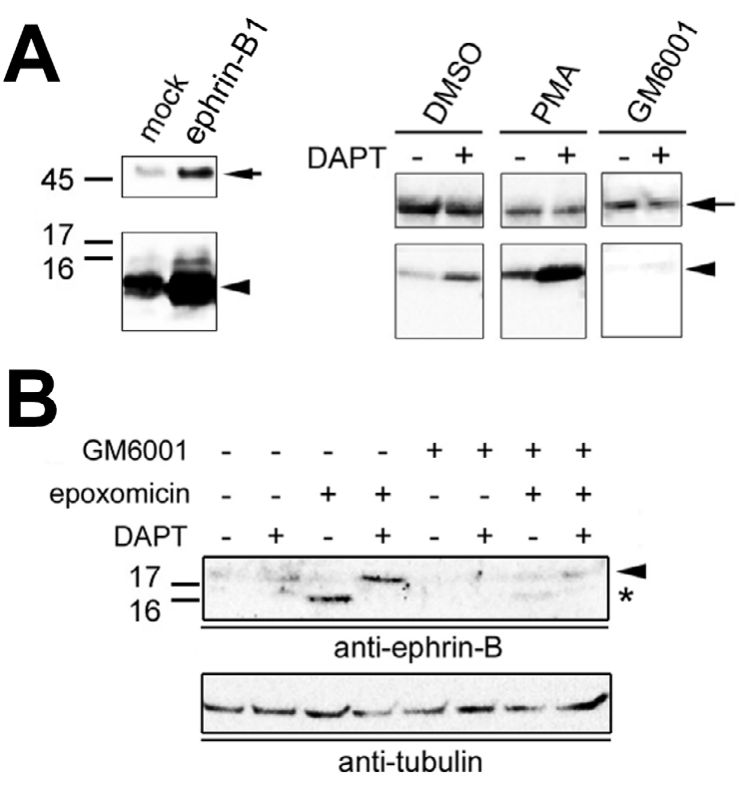
**Analysis of proteolytic processing of ephrin-B1**. A) Immunoblot analysis of COS cells overexpressing ephrin-B1 (*arrows*). ephrin-B1-CTF (*arrowheads*) was increased by DAPT and PMA treatment, whereas GM6001 completely diminished the ephrin-B1-CTF. B) Effect of protease inhibitors on endogenous ephrin-B CTF. Note that proteasome inhibition by epoxomicin caused an accumulation of CTF (*arrowhead*) and ICD (*asterisk*), that was abolished by GM6001 treatment.

**Figure 3:**
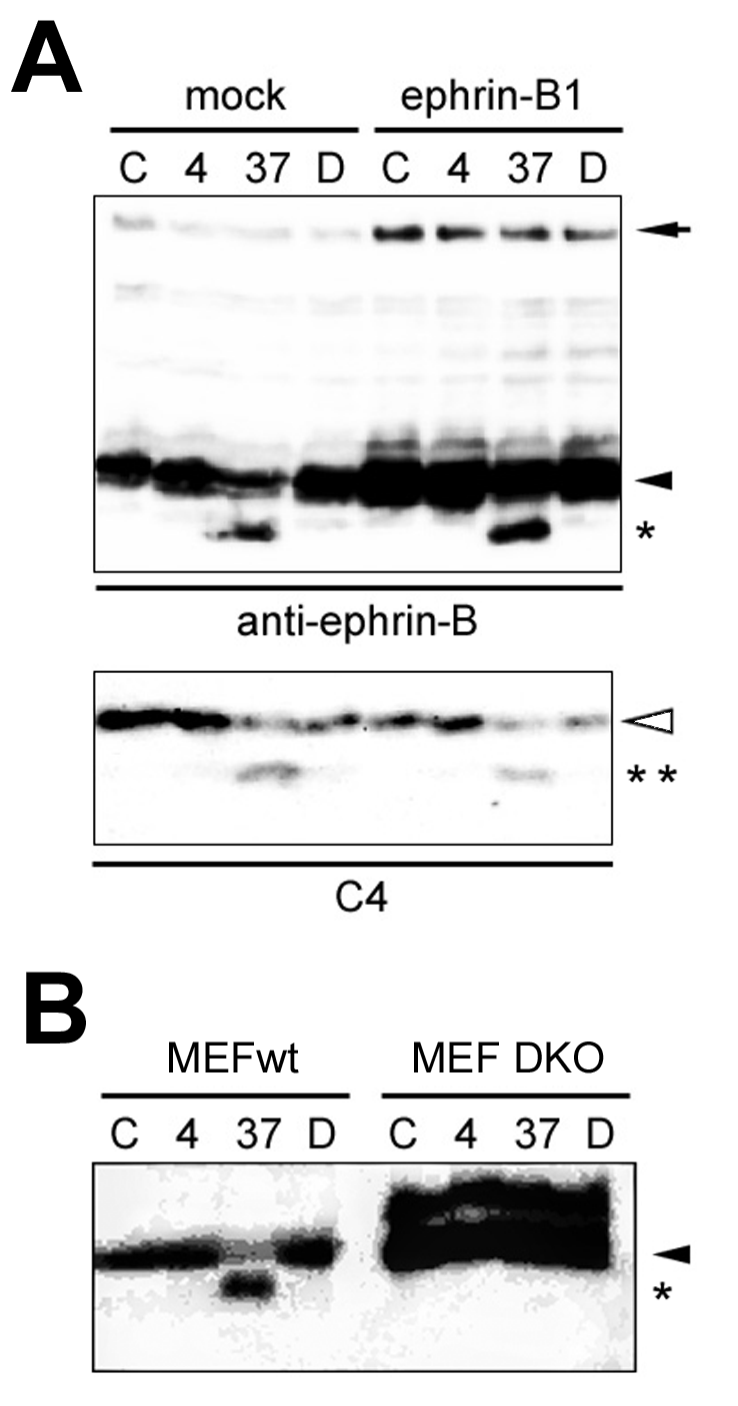
**Cell-free γ-secretase assay for ephrin-B1 cleavage**. A) Cell-assay using membranes from mock- or ephrin-B1-transfected COS cells. Samples incubated at 4 degree (4), 37 degree (37) or 37 degree with DAPT (D) were analyzed by immunoblotting by anti-ephrin-B antibody (*Upper panel*). Same fractions without incubation were indicated as "C". ephrin-B1 FL, ephrin-B-CTF and *de novo *generated ICD were shown by *black arrow, arrowhead *and *asterisk*, respectively. Endogenous APP (*Lower panel*) C-terminal stubs (*white arrowhead*) were cleaved by γ-secretase to generate AICD (*double asterisks*). B) Cell-free assay of membranes from wild-type MEF (MEFwt) and MEF lacking *Psen1 *and *Psen2 *(DKO). ephrin-B-CTF and *de novo *generated ICD were indicated by *arrowhead *and *asterisk*.

γ-Secretase cleaves membrane-tethered CTF within TMD to liberate N-terminal soluble peptides and C-terminal ICD after the processing by sheddase within the luminal region [3]. Treatment by phorbol esters has been reported to induce ectodomain shedding by activation of ADAMs (a disintegrin and metalloprotease) (e.g., tumor necrosis factor-α converting enzyme) or matrix metalloproteases (MMPs) [7,8]. Importantly, GPI-anchored ephrin-A2 is associated with TACE and cleaved upon binding with EphA receptor [9]. Consistent with the prediction above, overexpressed ephrin-B1 FL was cleaved to generate ~14–17 kDa CTF, that was increased by treatment with phorbol 12-myristate 13-acetate (PMA) and further augmented by preincubation with DAPT (Fig. 2A). Moreover, coincubation with GM6001, a specific MMP inhibitor, abolished the accumulation of ephrin-B-CTF, suggesting that ephrin-B FL is shed by matrix metalloprotease within the luminal region. However, ephrin-B-ICD, the final product of γ-cleavage, was hardly detected even in lysates of PMA-treated ephrin-B-overexpressing cells. Several reports indicated that γ-secretase-generated ICDs are hardly detected *in vivo *because of their short half-lives due to proteasomal degradation [3]. Consistent with this feature, treatment by epoxomicin, a potent irreversible proteasome inhibitor, caused an accumulation of ~10–12 kDa polypeptides (fig. 2B). These smaller bands disappeared upon treatment by DAPT and/or GM6001, suggesting that the generation of ~10–12 kDa bands is mediated by MMP and γ-secretase activity. Intriguingly, coincubation with DAPT and epoxomicin caused a marked accumulation of ephrin-B-CTF, which was abolished by GM6001, indicating that a fraction of ephrin-B-CTF undergo proteasomal degradation. Finally, incubation of membrane fractions from COS cells at 37 degree caused *de novo *γ-secretase-dependent generation of ~10–12 kDa bands in a similar manner to that of APP ICD (AICD) (Fig. 3A) [10,11]. These ~10–12 kDa bands were never generated from membranes of mouse embryonic fibroblasts (MEF) lacking *Psen1/Psen2 *(DKO) (Fig. 3B). Taken together, these data suggest that endogenous ephrin-B FL (~40–50 kDa) is constitutively processed by MMP within luminal region to generate CTF (~14–17 kDa), subsequently cleaved by γ-secretase and liberates ICD (~10–12 kDa), the latter being rapidly degraded by proteasome in cytoplasm.

### PS-dependent γ-secretase cleavage of ephrin-B1

**Figure 4:**
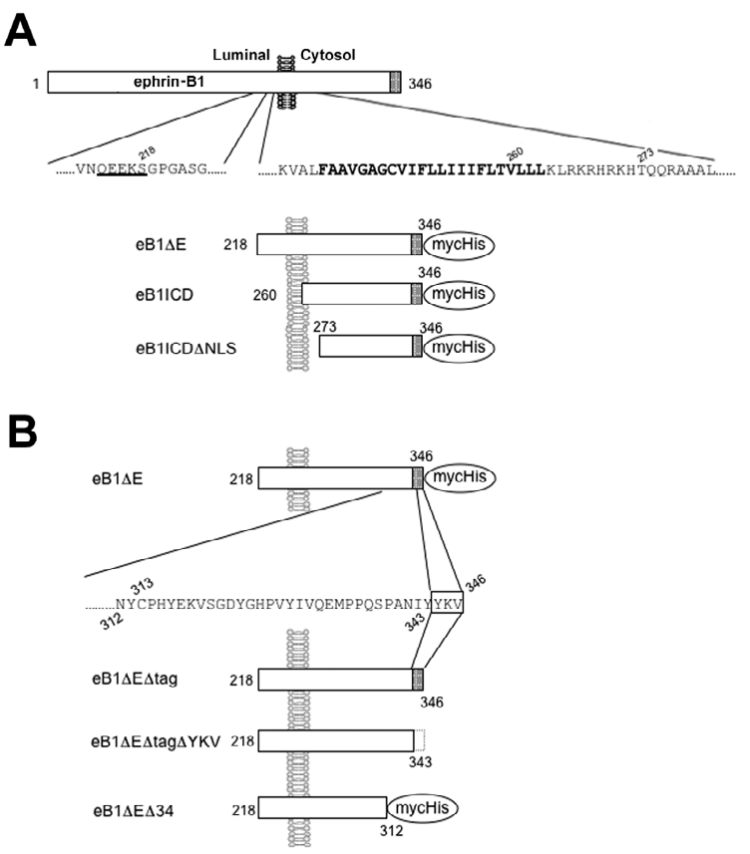
**Schematic depiction of ephrin-B1 derivatives used in this study**. A) N-terminally truncated ephrin-B1 derivatives. B) C-terminally truncated eB1ΔE derivatives. Putative amino acid sequence recognized by sheddase is *underlined*. Transmembrane domain is shown in *bold*. PDZ domain binding region (YKV) and Myc/His tag are indicated by *shaded box *and *oval*, respectively. *Numbers *indicate the residues in ephrin-B1 FL protein.

**Figure 5:**
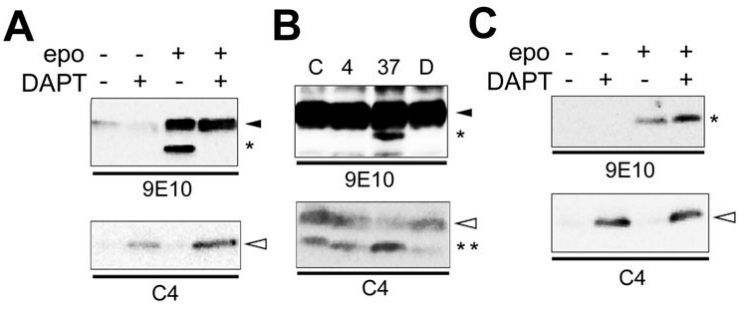
**Proteolytic processing of overexpressed ephrin-B1 derivatives**. A) Proteasome and γ-secretase inhibitor ("epo" and "DAPT", respectively) treatments on cells expressing eB1ΔE (*black arrowhead*). Proteolytically-generated ICD was shown by *asterisk*. DAPT inhibited the processing of eB1ΔE as well as endogenous APP (accumulation of C-terminal stub, *white arrowhead *in *lower panel*). B) Cell-free assay using COS membrane expressing eB1ΔE. *de novo *generated ICDs from eB1ΔE (*black arrowhead*) and APP C-terminal stub (*white arrowhead*) were indicated by *asterisk *and *double asterisks*, respectively. C) Inhibitor treatments (same as *A*) on cells expressing eB1ICD (*asterisk*). Accumulation of C-terminal stub (*white arrowhead*) by DAPT was shown at *lower panel*.

To further characterize the intramembrane proteolysis of ephrin-B by γ-secretase, truncated forms of ephrin-B1 fused with myc/His tag at N and C terminus were analyzed (Fig. 4A). eB1ΔE, that corresponds to CTF of ephrin-B1 starting at Ser^218 ^residue fused with a signal peptide, was expressed as a 20 kDa protein, and its protein level was increased by DAPT treatment, whereas no smaller bands were detected (Fig. 5A). However, the accumulation of a smaller ~17 kDa polypeptide was detected upon epoxomicin treatment, which was abolished by coincubation with DAPT. Furthermore, *de novo *γ-secretase-dependent generation of ICD-like smaller peptides was detected in a cell-free γ-secretase assay using membranes of cells transfected with eB1ΔE (Fig. 5B). Next we constructed a cDNA encoding eB1ICD, that corresponds to a predicted γ-secretase product, starting at intramembranous Val^260 ^residue, the latter being closest to the analogous cleavage site of the previously reported ICDs (e.g., Notch [12]), and C-terminally fused to a myc/His tag (Fig. 4A). eB1ICD was detected only after epoxomicin treatment, but not affected by DAPT (Fig. 5C). Thus, it seems reasonable to speculate that ICD of ephrin-B is constitutively and directly generated from truncated eB1ΔE by γ-secretase-mediated cleavage, but is highly labile because of its liability to proteasomal degradation.

**Figure 6:**
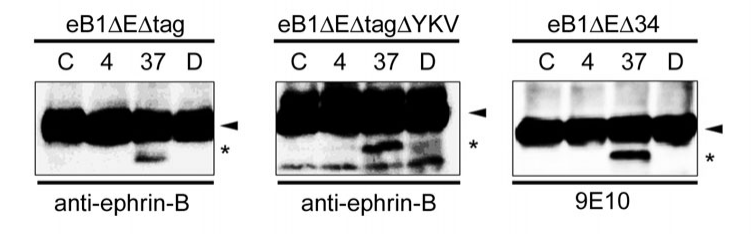
**Proteolytic processing of overexpressed eB1ΔE derivatives**. Cell-free assay using COS membranes expressing eB1ΔEΔtag (left), eB1ΔEΔtagΔYKV (middle) and eB1ΔEΔ34 (right). Antibodies used were indicated below the panels. All derivatives (*arrowheads*) were cleaved to generate ICD-like peptides (*asterisks*) by γ-secretase activity.

Truncated ErbB-4 receptor tyrosine kinase is a direct substrate for γ-secretase activity. PDZ domain binding motif at C terminus of ErbB-4 is suggested to be required for the intramembrane proteolysis [13]. Ephrin-B1 also harbors a PDZ domain binding motif consisting of Tyr-Lys-Val residues, that is thought to be crucial for the localization and function of ephrin-B1 through protein-protein interaction [14,15]. Furthermore, conserved tyrosines that are phosphorylated in response to stimulation by their ligands (i.e., EphB receptors) play an important role(s) in reverse signaling through phosphotyrosine-binding proteins [16-19]. To examine the role of these functional motifs at C terminus of ephrin-B1 in γ-secretase-mediated cleavage, we generated a series of C-terminally truncated eB1ΔE cDNAs: eB1ΔEΔtag that corresponds to ephrin-B-CTF without C-terminal myc/His tag, eB1ΔEΔtagΔYKV that lacks the tag as well as the PDZ domain binding motif, and eB1ΔEΔ34 that is deleted at the C-terminal 34 amino acids including all conserved tyrosines and fused with a C-terminal tag (Fig. 4B). Cell free assay using membrane fractions from transfected cells revealed that all the C-terminally modified eB1ΔE derivatives were cleaved by γ-secretase to generate ICD-like peptides (Fig. 6). These data suggest that these protein-protein interaction motifs at the C terminus of ephrin-B1 are dispensable for the γ-secretase-mediated proteolysis.

**Figure 7:**
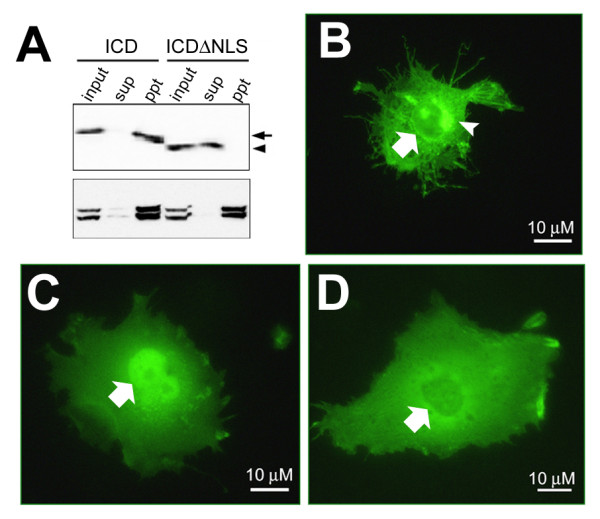
**eB1ICD translocated into the nucleus**. A) Immunoblot analysis of total (input), Triton-soluble (sup) or -insoluble (ppt) lysates from COS cells expressing eB1ICD (*arrow*) or eB1ICDΔNLS (*arrowhead*) using anti-myc (*upper panel*) or anti-lamin A/C (*lower panel*). B), C) and D) were the representative results of immunocytochemical analysis of COS cells expressing eB1ΔE, eB1ICD and eB1ICDΔNLS, respectively, using anti-myc antibody. Nucleus and golgi area were indicated by *arrows *and *arrowhead*, respectively.

γ-Secretase cleavage generates ICDs that in turn translocate into the nucleus mediated by the nuclear localization signals (NLS) and/or binding proteins [12]. Several ICDs are shown to form transcriptionally active complex to facilitate the transcription of downstream genes (e.g., AICD, NICD, CD44ICD) [3,5]. While there is no apparent canonical NLS in the cytoplasmic domain of ephrin-B, a basic amino acid cluster is located adjacent to the cytosolic face. As the basic amino acid cluster is known to function as NLS, we examined the fractionation analysis of cell lysates transfected with eB1ICD or eB1ICDΔNLS starting at Thr^273 ^residue (Fig. 4A). eB1ICD polypeptides accumulated in the Triton X-100-insoluble fraction containing lamin A/C, whereas eB1ICDΔNLS was detected only in soluble fraction (Fig. 7A). Immunofluorescence analysis revealed that eB1ICD was chiefly localized within nucleus as well as in the cytoplasmic region, whereas the immunoreactivity of eB1ICDΔNLS was limited to the cytoplasm (Fig. 7C,D). In contrast, eB1ΔE localized at cell membranes including Golgi area (Fig. 7B). These data suggest that ICD of ephrin-B enters the nucleus utilizing the basic amino acid cluster at the juxtamembrane region as NLS. Collectively, our findings suggest that ephrin-B1 is a genuine γ-secretase substrate, similarly to other authentic substrates like Notch.

### Membrane-attached truncated ephrin-B1 induced numerous cellular protrusions

**Figure 8:**
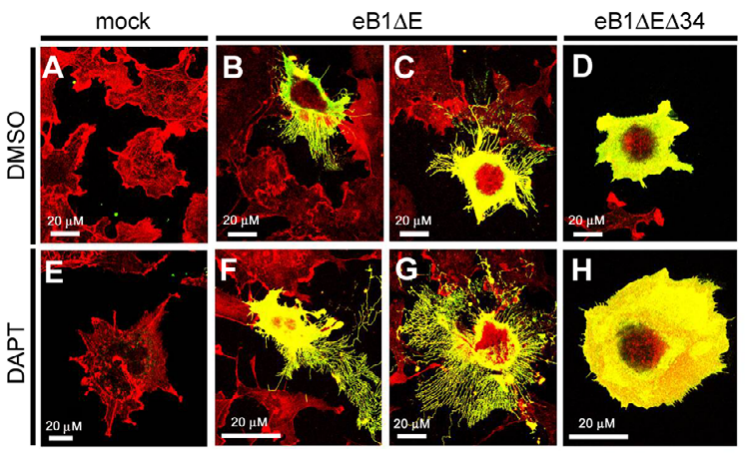
**Immunocytochemical analysis of COS cells expressing eB1ΔE and eB1ΔEΔ34**. COS cells were transfected with mock (A, E), eB1ΔE (B, C, F, G) or eB1ΔEΔ34 (D, H) and treated with DMSO (A-D) or DAPT (E-H). Fixed cells were probed with anti-myc (Green) and Rhodamin-Phalloidin (Red) for eB1ΔE and F-actin, respectively.

Ephrin-B is involved in the cell-cell contact-mediated signaling in collaboration with Eph receptors (e.g., cell migration and repulsion, neuritogenesis, angiogenesis) [14,15]. Recent data implicated ephrin-B in the morphogenesis of cells by regulation of actin dynamics through its phosphorylation and binding of partner proteins in cytoplasmic region [16-20]. To characterize the functional impact of γ-secretase-mediated cleavage in the ephrin-B1 signaling with reference to actin polymerization, we analyzed the morphology of COS cells transfected with ephrin-B1 derivatives. Intriguingly, several filopodia-like cellular processes that were highly enriched in actin filaments protruded from cells transfected with eB1ΔE (Fig. 8). The total length of cellular processes was increased by DAPT treatment (1.4 fold, p < 0.05). In contrast, overexpression of eB1ICD did not elicit cellular protrusions (Fig. 7C). Importantly, eB1ΔEΔ34 also did not enhance the generation of cellular protrusions, suggesting that the most C-terminal region of ephrin-B that encompasses conserved tyrosines is indispensable for the induction of actin polymerization. These data suggest that eB1ΔE regulates the actin dynamics of subplasmalemmal domain, that is negatively regulated by the γ-secretase activity-mediated cleavage.

## Discussion

Ephrin-B is a transmembrane protein that is implicated in bidirectional intercellular signaling in cooperation with its cognate receptor tyrosine kinase, Eph receptor [14,15]. Eph-ephrin binding induces the "forward signaling" through activation of the tyrosine kinase domain of Eph receptor. Moreover, ephrins also act as a receptor for Eph and recruit signaling into the host cell, which is referred to as the "reverse signaling". The cytoplasmic domain of ephrin-B ligands, that contains conserved tyrosine residues, mediates the phosphorylation-dependent and -independent reverse signaling, in cooperation with its binding proteins in FL form. Here, we show that ephrin-B is endoproteolyzed by metalloprotease (e.g., MMP) and then by γ-secretase. We also found that N-terminally truncated ephrin-B2 also is the substrate for γ-secretase (data not shown), suggesting that this proteolytic process is a common metabolic pathway for ephrin-B family proteins. Finally, membrane-tethered cytoplasmic domain of ephrin-B1 facilitated the formation of cellular protrusions enriched in polymerized actin, that is negatively regulated by γ-secretase activity. The liberated ICD translocated into the nucleus. Our findings suggest that ephrin-B-mediated proteolysis-dependent signaling is regulated by the γ-secretase activity.

Several transmembrane proteins including kinase-type receptors are now known to undergo γ-secretase cleavage after ectodomain shedding [3,5]. We screened novel substrates of γ-secretase using several criteria that are common to known substrates. Although the proteolytic processing of ephrin-B has not been reported, ephrin-A2, GPI-anchored type ephrin ligand, is cleaved by ADAM10/Kuzbanian upon activation by its cognate receptor, and this process is implicated in Eph-ephrin-A mediated axonal repulsion [9]. Our finding that ephrin-B is shed by metalloprotease suggest that Eph-ephrin-B interaction-mediated repulsion might also be regulated by proteolysis, in a similar manner to Notch [3]. However, recent reports showed that ephrin-B-mediated repulsion is regulated by trans-endocytosis after engagement with EphB receptor [19,20]. Furthermore, we observed that treatment of ephrin-B expressing cells with clustered EphB2 fusion protein had no effect on shedding as well as γ-secretase-mediated cleavage of ephrin-B (data not shown). We also found that ephrin-B stub undergoes γ-secretase-dependent intramembrane cleavage independently of its cytoplasmic C terminus, that contains a PDZ domain binding motif and conserved tyrosines. These findings suggest that γ-secretase cleavage of ephrin-B is not regulated by a protein-protein interaction within its C-terminal region. These data also indicate that the proteolytic processing of ephrin-B represents a constitutive signaling or metabolic pathway, that may be independent of the interaction with EphB2 receptor in COS cells. However, it has been shown that ephrin-B can interact with other EphB receptors including EphA4. Further characterization using a complete set of Eph receptors and/or cell lines is needed for the elucidation of the precise role of proteolytic processing of ephrin-B in its signaling.

To date, functions of γ-secretase-generated ICD as transcriptional activator (i.e., Notch, APP, CD44) or repressor (i.e., Jagged, N-cadherin) within nucleus have been reported [3]. We found that proteolytically generated eB1ICD, that is highly labile, localizes to the nucleus. Moreover, deletion of the acidic amino acid stretch located at the cytosolic face diminished the nuclear localization of this intracellular fragment, inferring its role as a nuclear transcriptional regulator. Although eB1ICD lacks known transactivation domain, intracellular domains of a subset of γ-secretase substrates interact with co-transcriptional activators after cleavage to facilitate nuclear translocation and/or transcriptional regulation (i.e, RBPJk for Notch [21], Fe65 for APP [22], YAP for ErbB-4 [23]). Further proteomic and genetic approach will be needed to clarify if eB1ICD regulates gene transcription.

Membrane-tethered eB1ΔE induced protrusion of a numerous actin-rich cellular processes. Most importantly, DAPT treatment increased the total length of these protrusions induced by expression of eB1ΔE. From these data, it is reasonable to speculate that the ephrin-B1-stub after ectodomain shedding may gain an activity to promote actin polymerization, that is subsequently down-regulated by γ-secretase-mediated cleavage. It is well known that Eph-ephrin-B signaling controls cell motility and adhesion through actin polymerization/depolymerization [14,15]. Furthermore, Eph-ephrin-B interaction mediates trans-endocytosis/retraction locally at contact sites between receptor cells and ligand cells, in a manner dependent on actin polymerization by Rac-signaling in receptor cells [16-20]. Several actin binding/regulating proteins (e.g., Grb4) were implicated in the regulation of actin dynamics through association with phospho-tyrosines in ephrin-B ICD [16,17,24-26]. Consistent with this, eB1ΔEΔ34, that lacks the Grb4-binding region, failed to promote protrusion of processes, and DAPT treatment without overexpression of eB1ΔE had no effect on cellular morphology. Intriguingly, genetic analysis revealed that several mutations in human ephrin-B1 gene are linked to familial and sporadic forms of craniofrontonasal syndrome (CFNS) [27,28]. As ephrin-B1 knockout mice showed similar defects in skeletal patterning [29,30], these CFNS-linked mutations are most likely leads to a loss-of-function of ephrin-B1. Of note, some CFNS-linked mutations cause a frameshift at the C terminus resulting in a loss of Grb4 binding site of ephrin-B1 [31]. Thus, the most C-terminal region of ephrin-B1 is indispensable for ephrin-B signaling, to which γ-secretase activity might act as a negative regulator. This negative regulation of ephrin-B signaling mediated by γ-secretase activity is very similar to what has been recently reported for deleted in colorectal cancer (DCC) in synaptic function [32].

While several transmembrane proteins are reported as substrate for γ-secretase [5], little information is available for the physiological significance *in vivo *of the intramembrane cleavage of these substrates other than Notch [4]. However, considering the fact that Eph-ephrin-B signaling shows functional synergy with Notch-Delta/Jagged signaling in somitegenesis [33,34] and vasoculogenesis [35], γ-secretase might be an important signaling component in these bi-directional signaling at multiple points. Our findings on the proteolytic processing of ephrin-B provide information about adverse side effects of γ-secretase inhibitors for the treatment of Alzheimer disease, as well as a novel direction for understanding the molecular mechanism of Eph-ephrin-B signaling.

## Materials and methods

### Construction of expression plasmid

A full-length cDNA encoding human ephrin-B1 was subcloned into a pcDNA3.1/Hyg vector (Invitrogen) from I.M.A.G.E clone (clone ID 3867060) (Invitrogen). cDNAs encoding eB1ΔE, eB1ΔEΔtag, eB1ΔEΔYKV, eB1ΔEΔ34, eB1ICD and eB1ICDΔNLS were amplified by PCR using following primer pairs: 5'-AGCTTGAGTGGCCCAGGTGCA-3' as a sense primer and 5'-GAATCCGAGACCTTGTAGTAGAT-3' as an antisense primer for eB1ΔE, 5'-TACTACAAGGTCTGATCGGATCCAAGCTTGTGTGGTGGAATTCTG-3' as a sense primer and 5'-CAGAATTCCACCACACAAGCTTGGATCCGATCAGACCTTGTAGTA-3' as an antisense primer for eB1ΔEΔtag, 5'-GCGAACATCTACTGATCGGATCCAAGCTTGTGTGGTGGAATTCTG-3' as a sense primer and 5'-CAGAATTCCACCACACAAGCTTGGATCCGATCAGTAGATGTTCGC-3' as an antisense primer for eB1ΔEΔYKV, 5'-CCCAAGCTTGAGTGGCCCAGGTGCA-3' as a sense primer and 5'-GGGGGATCCGAGTTGTTCTCTGTAGT-3' as an antisense primer for eB1ΔEΔ34, 5'-GGTACCACCATGGTCCTACTACTG-3' as a sense primer and 5'-GGATCCGACCTTGTAGTAGAT-3' as an antisense primer for eB1ICD, 5'-CCCGGTACCACCATGACACAGCAGCGGGCG-3' as a sense primer and 5'-GGGGGATCCGACCTTGTAGTAGAT-3' as an antisense primer for eB1ICDΔNLS.

### Cell culture, transfection and retroviral infection

Cell lines including SV40-transformed mouse embryonic fibroblasts (MEF) derived from wild-type (wt) or *PSen1^-/-^PSen2*^-/- ^(DKO) littermates (provided by Dr. B. De Strooper) were maintained as described [6]. Transient transfections of cDNAs into cells were performed by DEAE-dextran method (for COS cells), or using lipofectAMINE (Invitrogen) according to manufacturer's instructions [36]. Retroviral infection was performed as previously described [37]. To analyze the effect of protease inhibitors (i.e., *N*-[*N*-(3,5-difluorophenacetyl)-L-alanyl]-(*S*)-phenylglycine *t*-butyl ester (DAPT) (kindly provided by Drs. T. Kan and T. Fukuyama, The University of Tokyo [38]), GM6001 (Chemicon) and epoxomicin (SIGMA)) on the metabolism of ephrin-B and its derivatives, COS cells were cultured in DMEM in the presence of various concentrations of inhibitors for 24 hr and harvested.

### Cell-free γ-secretase assay

Membrane pellets were prepared as previously described and stored at -80 degree until use [39-42]. All procedures were performed at 4 degree. Membrane pellets were resuspended in 1 × γ buffer (-C) (10 mM HEPES, pH7.4, 150 mM NaCl, 10% glycerol, 5 mM EDTA, 5 mM 1,10-phenanthroline, 10 μg/ml phosphoramidon, Complete protease inhibitor cocktail (Roche Biochemicals)) with or without indicated inhibitors at 4 or 37 degree for 6–16 hrs. Control reactions were performed in the presence of 1% DMSO. The reaction was stopped by adding the sample buffer and boiling for 2 min. For the detection of *de novo *generated products, samples were separated by SDS-PAGE and analyzed by immunoblotting.

### Reagents and immunochemical analyses

Antibodies and reagents were purchased from Cell Signaling Technology (anti-myc 9B11), CHEMICON International (anti-lamin A/C), Molecular Probes (Alexa Fluor 488 anti-mouse IgG or Rhodamin-Phalloidin), Roche applied sciences (anti-c-myc 9E10), Santa Cruz (anti-ephrin-B1(C-18)) or SIGMA (anti-α-tubulin DM1A). Anti-G1L3 against PS1 loop region was described previously [40]. Anti-APP antibody C4 is a gift from Dr. Y. Ihara (The University of Tokyo). For preparation of Triton X-100 soluble/insoluble fractions, cells were directly lysed in HEPES buffer containing 1% Triton X-100 and centrifuged at 100,000 × g for 1 hr. Membrane preparation, immunoblot or immunocytochemical analyses were performed as previously described [36,37,39-42].

### Cell morphology assay

Transfected COS cells were replated on poly-L-lysine coated coverslips. After staining and mounting, images were taken by CCD camera or FV300 Fluoview conforcal microscopy (Olympus), and processed by Image J software. We defined "cells extending protrusions" by the number (>10) and the length (of the longest process that is longer than 1/3 of the diameter of cell body) of processes.

## Abbreviations

Abbreviations: Aβ, amyloid β-peptide; AD, Alzheimer's disease; AICD, APP intracellular domain; APP, amyloid-β precursor protein; CFNS, craniofrontonasal syndrome; CTF, carboxyl-terminal fragment; DAPT, {*N*-[*N*-(3,5-difluorophenacetyl)-L-alanyl]-*S*-phenylglycine *t*-butyl ester; DKO, *PSen1*^-/-^*PSen*2^-/-^double knockout mice; ELISA, enzyme-linked immunosorbent assay; FL, full-length; ICD, intracellular domain; MEF. Mouse embryonic fibroblast; NICD, Notch intracellular domain; NTF, amino-terminal fragment; mt, mutant; PMA, phorbol 12-myristate 13-acetate; PS, presenilin; TMD, transmembrane domain; wt, wild-type

## Authors' contributions

TT contributed to the conception, design, analysis and interpretation of the data and was responsible for manuscript preparation. ST carried out the experiments described in this article and contributed to the preparation of data for publication. YM interpreted the data and aided in experimental design. TI was responsible for manuscript preparation. All authors read and approved the final manuscript.
